# Supplementary material for: An automated alarm system for food safety by using electronic invoices
Source: PLoS One. 2020 Jan 24;15(1):e0228035. doi: 10.1371/journal.pone.0228035 (PMC6980643; doi:10.1371/journal.pone.0228035)
Supplement: Attachment — Submitted filename: Response to Reviewers 20191119.docx [file pone.0228035.s001.docx]

**Journal Requirements:**

1. When submitting your revision, we need you to address these additional requirements.

 Please ensure that your manuscript meets PLOS ONE's style requirements, including those for file naming. The PLOS ONE style templates can be found at

<http://www.journals.plos.org/plosone/s/file?id=wjVg/PLOSOne_formatting_sample_main_body.pdf> and <http://www.journals.plos.org/plosone/s/file?id=ba62/PLOSOne_formatting_sample_title_authors_affiliations.pdf>

**Reply:**

We revised the manuscript according to PLOS ONE's requirements.

2.  Thank you for stating the following in the Acknowledgments Section of your manuscript: "The work was funded by MOST 106-2314-B-O 18-001-MY2
from the Ministry of Science and Technology. The e-invoice data was provided by
Fiscal Information Agency of Taiwan Ministry of Finance."

We note that you have provided funding information that is not currently declared in your Funding Statement. However, funding information should not appear in the Acknowledgments section or other areas of your manuscript. We will only publish funding information present in the Funding Statement section of the online submission form.

Please remove any funding-related text from the manuscript and let us know how you would like to update your Funding Statement. Currently, your Funding Statement reads as follows:  The author(s) received no specific funding for this work.

**Reply:**

We remove funding-related text from the manuscript and update the Funding Statement.

“The work was funded by MOST 106-2314-B-018-001-MY2 from the Ministry of Science and Technology. The funders had no role in study design, data collection and analysis, decision to publish, or preparation of the manuscript. The e-invoice data was provided by Fiscal Information Agency of Taiwan Ministry of Finance. We also like to thank Changhua County Public Health Bureau for the constructive discussion.”

3.  Please could you provide further details about the expert-knowledge based process in section 2.2., step 3:
*If the 'researchers' involved were not the authors, please provide details of their positions and expertise and how they were recruited for this exercise.
*Please clarify if you obtained consent from the researchers to participate in this study.
*Please provide details of how the process was carried out: What questions were the researchers asked? How did they come to an agreement?

**Reply:**

1. All the researchers who involved in data analysis and paper writing were authors.
2. Process: (I) The question “how to efficiently alert food safety issues by using e-invoice” was raised by one of the authors (Dr Yeh), who has served as Director-general of Changhua County Health Bureau for years, and has rich experience in dealing with the food safety issues with related manufacturers. (II) The authors had also hold two meetings with some manufacturer managing teams to discuss about the proper on identifying irrational transaction. (III) the correspondent author (Dr Lian) had signed an agreement with Fiscal Information Agency (FIA) of the Ministry of Finance to access the e-invoice data, and analyze it under the supervision of FIA.

On 'Data processing' section, we added:

“A bilateral agreement was signed by FIA and the institute of the authors (National Changhua University of Education) for permission of accessing the e-invoice data and analyzing it under the supervision of FIA. “

“The dataset used in this study is owned by FIA, and was analized inside the FIA data center. To access the data researchers can contact Taiwan FIA to apply for the authorization.”

On page 11 we added:

“The authors had held several consultation meeting with experts including the Director-general of Changhua County Health Bureau, who has rich experience in dealing with the food safety issues, and managing teams from several A-manufacturers, to discuss the proper features on classifying the "rational" vs "irrational" transactions.”

**Reviewers' comments:**

Reviewer's Responses to Questions

Comments to the Author

1. Is the manuscript technically sound, and do the data support the conclusions?

The manuscript must describe a technically sound piece of scientific research with data that supports the conclusions. Experiments must have been conducted rigorously, with appropriate controls, replication, and sample sizes. The conclusions must be drawn appropriately based on the data presented.

Reviewer #1: Partly

Reviewer #2: Yes

________________________________________

2. Has the statistical analysis been performed appropriately and rigorously?

Reviewer #1: I Don't Know

Reviewer #2: Yes

________________________________________

3. Have the authors made all data underlying the findings in their manuscript fully available?

The PLOS Data policy requires authors to make all data underlying the findings described in their manuscript fully available without restriction, with rare exception (please refer to the Data Availability Statement in the manuscript PDF file). The data should be provided as part of the manuscript or its supporting information, or deposited to a public repository. For example, in addition to summary statistics, the data points behind means, medians and variance measures should be available. If there are restrictions on publicly sharing data—e.g. participant privacy or use of data from a third party—those must be specified.

Reviewer #1: Yes

Reviewer #2: No

________________________________________

4. Is the manuscript presented in an intelligible fashion and written in standard English?

PLOS ONE does not copyedit accepted manuscripts, so the language in submitted articles must be clear, correct, and unambiguous. Any typographical or grammatical errors should be corrected at revision, so please note any specific errors here.

Reviewer #1: Yes

Reviewer #2: Yes

________________________________________

5. Review Comments to the Author

Please use the space provided to explain your answers to the questions above. You may also include additional comments for the author, including concerns about dual publication, research ethics, or publication ethics. (Please upload your review as an attachment if it exceeds 20,000 characters)

Reviewer #1: The paper presents a system to detect edible oil manufactures that are suspicious to sell adulterated oils. The system makes use of data mining techniques to detect anomalous individuals in a big sample.

The aim of the paper is interesting and potentially new. Nevertheless, I have seen several shortcomings in the paper, described below. I recommend the authors considering the comments in order to improve the quality of the paper:

1) The contribution is not contextualized. I miss a section dealing with related work about, at least, systems to detect anomalous individuals using data mining techniques. There are many recent contributions in this aspect, for example in financial fraud detection, which is related to this paper. By doing a literature review, the authors can put it clear what the paper adds to knowledge and practice in this line of research.

**Reply:**

Thank you for your suggestions.

We have added some paragraphs at the introduction section to describe the background of this study regarding food safety and big data. We have also dealt with related works about prediction models of food fraud using big data approach.

So it reads: “The emerging use of rapidly collected, complex data in unprecedented quantities is ushering the world into the era of big data [1]. Although utilization of big data has the potential to afford new insights, improve decision making and governance, and enhance the quality and efficiency of products and services, their application in the food safety domain is still limited [2]. Food safety data and information comprise structured and non-structured data from multiple sectors such as environment, animal, agriculture, food, public health, trade and economy. Previous efforts have explored the predictive power of big data in foodborne illness surveillance, environmental microbial contamination of crops, food safety violations and interpretation of genomic data for tracking and tracing foodborne illnesses [2-3].

In past decades, a variety of food fraud incidents have been reported in many countries. Such incidents have had a profound impact on public health and consumer confidence in the safety of food [4]. In response to these incidents, one of the main focuses of food fraud prevention has been on novel prediction models of food fraud using a big data approach, which considered different factors from within and outside the food supply chain. [5-8].”

2) In the Data section, I miss an explanation of how expert knowledge was extracted. Do you interview some key agents? How many? Which was the criterium to select the experts?

**Reply:**

One of the authors (Dr Yeh) has served as Director-general of Changhua County Health Bureau for years, and has rich experience in dealing with the food safety issues with related manufacturers. The authors had also hold two meetings with some manufacturer managing teams to discuss about the proper on identifying irrational transaction.

On page 11 we added

“The authors had held several consultation meeting with experts including the Director-general of Changhua County Health Bureau, who has rich experience in dealing with the food safety issues, and managing teams from several A-manufacturers, to discuss the proper features on classifying the "rational" vs "irrational" transactions.”.

3) The selection of the training data should be also discussed. Why not selecting data from a certain time period (e.g., one year) as training data and the following period as testing data? By doing so, the results would show the system ability to predict suspicious manufactures in the future.

**Reply:**

We used the 5-fold strategy with 8:2 training-testing-ratio. This is now mentioned on page 10.

We cannot divide the data into training and testing sets by year due to the fact that the compulsory law for using e-invoice started only in October 2014. Thus, several B-labeled manufacturers did not use it before then. After the scandals broke out, certain B-labeled manufacturers closed down, and the transaction records became unavailable.

4) Which are the specific features influencing the most on the classification? It would be interesting to know and interpret these features.

**Reply:**

Among the features we had tried, the combinations of X_1_ and X_2_ have relatively better performance:

$\text{X}_{1}=\text{log(U}_{\text{i}}/\text{D}_{\text{i}})$ $\text{X}_{2}=\text{log(U}_{\text{i}}/\text{D}_{\text{i-1}})$

X_1_ is the log-transformed ratio of sales to purchase in the same month i, and X_2_ considers the lag effect between purchasing materials and sale products. We added the following interpretation in Discussion on page 16-17:

“The excess large values of X_1_ , for example, X_1_ >6, indicates that the turnover of products sales is $e^{6}$ (>400) times more than that of material purchase for the specific manufacturer in the same month, while X_2_ take into account of the lag effect between product sale and materials purchasing from previous month. With both X_1_ and X_2_ >6, the manufacturer had sold products of value 400 times more than the materials it had purchased within a 2-month period. One possible interpretation for this scenario is that a large part of the materials used in products are not considered as proper ingredients for edible-oil manufacture. The increase of se for naïve decision tree can be achieved by lowering the threshold 6 to smaller value, however as a tradeoff this will reduce its sp resulting extra false alarm.”

5) Related to the previous comment, which would be the interpretation of the naïve decision tree in section 3.2? Do you have any explanation about why this rule identifies suspicious manufacturers?

**Reply:**

We had added the explanation as answered in last question.

6) Part of the stuff in the Discussion section (that dealing with figure 4(b)) would be better located in the Results section. I also miss the last column in Table 4, commented in the Discussion section.

**Reply:**

We had move the part that dealing with figure 4(B) to Results section.

We remove the words “the last column in Table 4”. The result already shown in Fig 4.

7) I also recommend including a Conclusion section.

**Reply:**

We added a Conclusion section in abstract.

Reviewer #2: I think the manuscript is very interesting and may be very useful to the government from Taiwan. In addition, the method may be inspiring for other countries.

I have some suggestions, as follows:

1. “Prevention is always better than remedy”- I think the sentence is very colloquial and should be replaced.

**Reply:**

The sentence had been replaced by

“The outbreak of major food safety incidence could result costly public panic and damage of goodwill [13].”

2. “Early detection of food safety events via warning signs of suspicious transactions is thus needed.” – Please give more details about it. Does the e-invoice was already used to detect food adulteration? What are the possible clues to detect a food safety adulteration using the e-invoice system?

Reply:

We rephrased the sentence and added a new paragraph on page 6 to explain:

“To establish traceability system, all the related records (ie. invoices, receiving and shipping papers etc.) of each transaction should be kept and retained for a period of five years at least, regardless of whether the form of such records is in paper, electronic or otherwise. To assess the consistency of these records and to identify unusual and inappropriate trends is time-consuming and demanding for experienced manpower because of the heavy burden to inspect the large amount of detailed information concerning date of purchase or supply, name of products, quantity received or supplied, and name and address of the suppliers or distributors etc.

Utilizing e-invoice big data provides an opportunity to overcome aforementioned difficulty. Early detection of food fraud incidents via warning signs of suspicious transactions is plausible approach.”

3. “All registered edible oil manufacturers with a capital amount of over 1 million USD in Taiwan have been required to use an e-invoice system” – Can you include the reference about this requirement?

**Reply:**

The reference was added on page 8 and in reference [19].

19. Ministry of Health and Welfare/Food Drug Association (MOHW/FDA). Taiwan Food and Drug Administration 2015 Annual Report, page 115. https://www.fda.gov.tw/tc/includes/GetFile.ashx?id=f636694230125946085 Accessed 10.26.19.

4. What is FIC? Please include the meaning of abbreviations on their first use.

**Reply:**

We corrected “FIC” to “FIA” and it was first time mentioned with full name on page 6.

FIA is the abbreviation of the Financial Intelligence Agency.

5. I think the methods and results sections could be more detailed. The method could be improved to increase the interest from food safety/ food policy specialists and stakeholders

**Reply:**

We had add the description of process, including consultation meetings with some manufacturers and agreement with FIA of the Ministry of Finance to access the e-invoice data, and analyze it under the supervision of FIA.

New paragraph about food safety/ food policy and consumer’s right were added in page 4-6 in Introduction as well as in Discussion (page 15-17) as requested by other comments.

6. I don’t think Table 1 is necessary. You could include the data in the text.

**Reply:**

The content of Table 1 is now included in the text (page 8).

7. “Researchers selected 60 keywords that they agreed could best distinguish edible oil-related items from others.” Can you give some examples?

**Reply:**

These keywords could be used for inclusion criteria like “Canola (kanola) oil”, “grapeseed”, “sunflower (seed)”, and “cold pressing” etc., or as exclusion like “sauce”, “flavor”, “cosmetic”, and “skin”, etc..

8. The discussion should be improved. Topics about food safety and consumer’s right could be included. Are there any other studies about using an automated system to detect frauds? The system implementation is expansive?

**Reply:**

We added an paragraph in discussion page 15:

“To establish traceability system, all the related records (ie. invoices, receiving and shipping papers etc.) of each transaction should be kept and retained for a period of several years, regardless of whether the form of such records is in paper, electronic or otherwise. To assess the consistency of these records and to identify unusual and inappropriate trends is time-consuming and demanding for experienced manpower because it is a heavy burden to inspect the large amount of detailed information concerning date of purchase or supply, name of products, quantity received or supplied, and name and address of the suppliers or distributors etc. Furthermore, since information provided by various suppliers differed with respect to food classiﬁcations, product categories and product naming conventions, few regulatory agencies can inspect all the players, upstream and downstream, in the entire food supply chain. Digitalized transactional big data that recorded users’ behavior had been applied for human health surveillance, for example, prediction of flu trends [24]. However, no literature had been found on applying electronic invoice for food safety.”

And on page 17:

“As modern food supply chains become more and more complex, the importance of food production transparency increases. Consumer expectation and right to know has added a challenging dimension to this transparency. Transparency in food supply chains via traceability requires implementation of new technologies such as the Internet of Things (IoT), blockchain and Big Data analytics [25-26]. In response to the food incidences in Taiwan since 2011 [10], Taiwan's Legislative Yuan passed the Act Governing Food Safety and Sanitation in 2013, and enacted new regulations in 2014 [9]. The amendment aim to provide information about food safety as consumers right with efficient trace management of materials, which is based on the registration of their importation, the labeling of additives, the labeling of final products, etc.. Beside that, it also important for government to be able to identify suspicious cases before becoming public event.”

9. “Prevention is always better than cure” – same as the sentence aforementioned

**Reply:**

The sentence had been replaced by

“The outbreak of major food safety incidence could result costly public panic and damage of goodwill [13].”

10. Only one reference is from 2017 and one from 2016. The other references are from <2015. I think more recent studies can be included. More references about food issues could be included.

**Reply:**

We now added the following reference.

- Wyber R, Vaillancourt S, Perry W, et al. Big data in global health: improving health in low- and middle-income countries. Bull World Health Organ. 2015; 93(3): 203–208.

doi: 10.2471/BLT.14.139022

- Marvin HJ, Janssen EM, Bouzembrak Y, Hendriksen PJ, Staats M. Big data in food safety: An overview. Critical Reviews in Food Science and Nutrition. 2017;57(11):2286-2295.

doi: 10.1080/10408398.2016.1257481

- Kannan V, Shapiro MA, and Bilgic M. Hindsight Analysis of the Chicago Food Inspection Forecasting Model. Presented at the AAAI Fall Symposium Series (FSS) 2019: Artificial Intelligence in Government and Public Sector. Arlington, Virginia, USA.
- Spink, J. and Moyer, D. C.. Defining the Public Health Threat of Food Fraud. Journal of Food Science.2011; 76(9):R157-63. doi: 10.1111/j.1750-3841.2011.02417.x.
- Fritsche J. Recent Developments and Digital Perspectives in Food Safety and Authenticity. Journal of Agricultural and Food Chemistry, 2018;66 (29), 7562-7567.doi: 10.1021/acs.jafc.8b00843
- Marvin HJP, Bouzembrak Y, Janssen EM, van der Fels-Klerx HJ, van Asselt ED, Kleter GA. A holistic approach to food safety risks: Food fraud as an example, Food Research International. 2016; 463-470. doi: 10.1016/j.foodres.2016.08.028, 89
- Bouzembrak Y, Steen B, Neslo R, Linge J, Mojtahed V, Marvin H.J.P. Development of food fraud media monitoring system based on text mining, Food Control. 2018; 93; 283-296.

doi: 10.1016/j.foodcont.2018.06.003

- Verhaelen, K., Bauer, A., Günther, F., Müller, B., Nist, M., Ülker Celik, B., et al. Anticipation of food safety and fraud issues: ISAR - a new screening tool to monitor food prices and commodity flows. Food Control. 2018;94; 93–101. doi: 10.1016/j.foodcont.2018.06.029
- Richterich, A.Using Transactional Big Data for Epidemiological Surveillance: Google Flu Trends and Ethical Implications of ‘Infodemiology’ . In: Mittelstadt B., Floridi L. (eds) The Ethics of Biomedical Big Data. Law, Governance and Technology Series. 2016;29: 41-72. Springer, Cham
- Astill J, Dara RA, Campbell M, Farber JM, Fraser E.D.G, Sharif S, et al. Transparency in food supply chains: A review of enabling technology solutions, Trends in Food Science & Technology. 2019; 91:240-247. [doi:10.1016/j.tifs.2019.07.024](https://doi.org/10.1016/j.tifs.2019.07.024).
- Messer KD, Costanigro M, Kaiser HM. Labeling Food Processes: The Good, the Bad and the Ugly, Applied Economic Perspectives and Policy. 2017;39(3): 407–427. doi:10.1093/aepp/ppx028
