# Supplementary material for: An automated alarm system for food safety by using electronic invoices
Source: PLoS One. 2020 Jan 24;15(1):e0228035. doi: 10.1371/journal.pone.0228035 (PMC6980643; doi:10.1371/journal.pone.0228035)
Supplement: Attachment — Submitted filename: Response to Reviewers.docx [file pone.0228035.s002.docx]

Dear Sir:

We had revised the paper according to reviewer’s comments item by item..

Iebin Lian

_________________________________________

6. Review Comments to the Author

Reviewer #1: The authors have followed some of my recommendations in the first review and now the method and results are clearer. Nevertheless, I need further insist in the first point of my previous review, about the contextualization of the contribution. The authors have framed the work in the literature around food fraud prevention using big data (references 5-8, 24-26). That’s fine, but simply mentioning some references in brackets does not help very much. Your study would be more valued if you make a comprehensive (not necessarily very long) review about the state of the art and what this paper adds to this body of research. In other words, I recommend commenting the cited references (perhaps including new ones), maybe in a new section of related work, highlighting the previous findings and what your study adds.

Reply:

We thanks you for the advises and had added some descriptions for the most recent literatures [3, 6-8] on page 4 and 5.

I have also some other minor comments:
1) Is there any error when citing references 16-17 and 18? It seems reference 16 should be reference 18.

Reply:

We corrected citing references 16-18.

2) Details about the number and role of experts consulted are better located in epigraph “Step 3”, page 9.

Reply:

Thank you for your suggestions.

We have added some paragraphs at “Step 3” about the number and role of experts consulted.

3) The quality of the figures should be much improved. I cannot read Fig 3 and Fig 4B.

Reply:

The separate files of figures have higher resolution than those converted to pdf.
